# Estimation of Full-Body Poses Using Only Five Inertial Sensors: An Eager or Lazy Learning Approach?

**DOI:** 10.3390/s16122138

**Published:** 2016-12-15

**Authors:** Frank J. Wouda, Matteo Giuberti, Giovanni Bellusci, Peter H. Veltink

**Affiliations:** 1 Institute for Biomedical Technology and Technical Medicine (MIRA), University of Twente, P.O. Box 217, Enschede 7500 AE, The Netherlands; p.h.veltink@utwente.nl; 2 Xsens Technologies B.V., Pantheon 6a, Enschede 7521 PR, The Netherlands; Matteo.Giuberti@xsens.com (M.G.); Giovanni.Bellusci@xsens.com (G.B.)

**Keywords:** inertial motion capture, orientation tracking, machine learning, neural networks, nearest neighbor search, human movement, reduced sensor set

## Abstract

Human movement analysis has become easier with the wide availability of motion capture systems. Inertial sensing has made it possible to capture human motion without external infrastructure, therefore allowing measurements in any environment. As high-quality motion capture data is available in large quantities, this creates possibilities to further simplify hardware setups, by use of data-driven methods to decrease the number of body-worn sensors. In this work, we contribute to this field by analyzing the capabilities of using either artificial neural networks (eager learning) or nearest neighbor search (lazy learning) for such a problem. Sparse orientation features, resulting from sensor fusion of only five inertial measurement units with magnetometers, are mapped to full-body poses. Both eager and lazy learning algorithms are shown to be capable of constructing this mapping. The full-body output poses are visually plausible with an average joint position error of approximately 7 cm, and average joint angle error of 7${}^{\circ }$. Additionally, the effects of magnetic disturbances typical in orientation tracking on the estimation of full-body poses was also investigated, where nearest neighbor search showed better performance for such disturbances.

## 1. Introduction

Human motion has been a research topic of interest in many fields for a long time. The increasing availability of high-quality motion capture systems [1,2,3,4] contributed to this topic, by allowing easier and more accurate three-dimensional human motion capturing [5]. The introduction of inertial motion capture systems, which do not rely on any external infrastructure, made full-body movement analysis feasible in an ambulatory setting [1]. These systems require sensors to be attached to each main body segment (e.g., 17 sensors in Xsens MVN [6]). By reducing the number of required body-worn sensors, such systems would be less obtrusive and the usability would improve, which could potentially lead to applications that require use in daily life. Another probable benefit would be the reduction in costs.

Many studies have proved that human movements contain redundant information and can be concisely described using fewer dimensions than the degrees of freedom of the human body [7,8,9]. This opens the way for human motion capture using a reduced set of sensors. The first approaches in this field used procedural models (based on empirical and biomechanical models, they offer less control but are not perceived as realistic [10]). Badler et al. [11] used four magnetic sensors (placed on the head, pelvis and both hands) and inverse kinematics to estimate upper body joint positions. Behavioral models were used for the estimation of the lower body joint positions, which resulted in the best estimation of gait poses. Another example of a heuristic-based system used eight magnetic sensors to estimate full-body movements by applying an analytical solution for the authors’ defined kinematic chains problem [12].

The increasing availability of motion capture data led to the use of data-driven approaches to deal with less information than provided by current full-body motion capture systems. One of the first data-driven approaches was presented by Chai and Hodgins [13], who used six reflective markers (captured with two video cameras) providing the position of anatomical landmarks to estimate full-body movements. A form of Nearest Neighbor Search (NNS) was used to map the lower dimensional input signals to full-body poses. The search space was limited with Principal Component Analysis to create a local linear model. The use of position-based features particularly fits with methods such as NNS, but calls for an external infrastructure (such as cameras) which limits the applicability to small (mostly indoor) areas.

In later years, Slyper and Hodgins [14] investigated a system composed of five accelerometers attached to the upper-body. Their results were promising and encouraged others to further investigate the use of such sensors for this problem. Tautges et al. [15] built upon that work by also using a few accelerometers, but with the addition of scaling in the temporal domain to also enable estimation of movements performed at different speeds. Riaz et al. [16] added ground contact information to the estimation framework of Tautges et al. [15] to estimate full-body poses using three accelerometers (placed on the wrists and lower back). These works showed the effort of moving towards an infrastructure-less setting, but the use of raw accelerometer data limited the potential performance of such methods, as these provide information of movement but not of a single pose, unlike position features.

The above-mentioned methods all adopted some form of NNS in their estimation framework. NNS belongs to the family of lazy learning algorithms [17], since no model is created during a training phase. Although these algorithms have been shown to be a good choice for the estimation of full-body movements using a reduced sensor set [13,14,15,18], the fact that the (typically large) training database needs to be stored makes them less appealing for real-time portable (or even embedded) applications. Even though smart search techniques (such as k-dimensional trees [13,15,19]) have been proposed to decrease search time, training databases cannot be indefinitely large to search and estimate output in real-time. Eager learning algorithms, opposed to lazy learning ones, do not have this requirement and a model is trained to concisely describe relationships between inputs and outputs in a training database. Liu et al. [20] applied a combination of both an eager and lazy learning approach to estimate full-body movements using six inertial and ultrasound sensors. Their approach combined previously estimated poses and their neighbors to construct a local linear model for prediction of the current pose. Starting from a similar setup, Kim et al. [21] used a kernel canonical-correlation-analysis-based regression. Although both works reported promising results with the use of eager learning approaches, their methods required several models to be trained at different time steps, thus increasing computational complexity. Moreover, both methods required position-based features and showed dependency on an external infrastructure.

A few examples of training global models for human movements can also be found in literature. For instance, support vector regression has been applied to the 2-Dimensional (2D) upper body pose estimation from images [22] and Artificial Neural Networks (ANNs) have been applied for the estimation of 3-Dimensional (3D) movements from 2D body poses [23]. None of these works, however, focus on estimating full-body movements from a reduced set of body-worn sensors.

In the efforts of developing a self-contained ambulatory system, preliminary work (chapter 5 of the PhD thesis) by one of the authors [24] has shown the feasibility of estimating full-body poses using an NNS-based approach with orientation features. However, this method also implemented simulated features, which would require additional sensor technologies. The choice for orientation features was mainly driven by the fact that current orientation tracking solutions [1], based on sensor fusion of inertial and magnetic data, have been proved to be highly effective at providing users with easy access to accurate and very informative quantities such as sensor orientations. Furthermore, orientations directly provide information of the current body pose, unlike only accelerations. In this work, we aim at further investigating the effectiveness of using orientation features in combination with a lazy learning (NNS) and an eager learning (ANN) algorithm. Since the main focus of the work is to investigate two learning paradigms (i.e., lazy and eager), the algorithm choice has been mainly driven by consistency with previous literature. Note that the choice between a lazy and eager learning approach shows a functional trade-off. Lazy learning, by not building any specific model, is more effective in preserving idiosyncrasies of training data. However, for the same reason, the computational complexity at run-time grows with the size of data. On the other hand, eager learning requires much longer training time, but the conciseness of the trained model makes it more appealing to real-time applicability. A data collection campaign has been carried out to create a large training database composed of movements from different subjects performing various activities and used to test the two algorithms. The sensor orientations provided by five Xsens motion trackers [6] (each containing an Inertial Measurement Unit (IMU) and magnetometer) have then been used as inputs to the learning algorithms to estimate full-body poses. Our method enables the realistic estimation of full-body poses using a reduced set of IMUs.

The remainder of this paper is organized as follows. In Section 2, we describe the data collection and processing. In Section 3, the detailed performance of both algorithms for different configurations and activities, and the effect of magnetic disturbances are described and discussed. Finally, conclusions of this work are presented in Section 4, and possible future work is described in Section 5.

## 2. Methods

### 2.1. Subjects

Six healthy subjects (three males and three females; age: 29.0 ± 11.9 years; height: 177.2 ± 7.2 cm; weight: 83.1 ± 11.1 kg; Body Mass Index (BMI): 26.5 ± 3.7 kg/m^2^; all dominant right-handed) volunteered to participate in the current study, for which ethical approval was obtained. Before participating in this research, each subject signed an informed consent form.

### 2.2. Experimental Protocol

Subjects were verbally instructed to perform different types of movements (ranging from gait, Activities of Daily Living (ADLs), to sports), as described in Table 1. The exact execution of these movements (namely, style and speed) was left to individual interpretation. Each trial was performed three times to guarantee large intra- and inter-subject variations in the movement database (which can be seen from some representative recordings shown in accompanying videos). Overall, approximately 25 min of motion capture data was recorded for each subject. The measurements were performed in the gait laboratory of Roessingh Research and Development (Enschede, the Netherlands). The working area used for data collection was chosen trying to minimize magnetic disturbances (e.g., large metal constructions were avoided and ferro-magnetic objects, when possible, were removed from the working area), such that good quality measurements were possible. MVN Studio offers some dedicated tools to measure magnetic field experienced in the environment and to test its homogeneity.

### 2.3. Instrumentation

The reference full-body motion capture system used in this study is Xsens MVN (Xsens Technologies B.V., Enschede, the Netherlands), in this manner, both learning approaches could be compared to the performance of an ambulatory motion capture system. Xsens MVN consists of a full-body Lycra equipped with 17 IMUs with magnetometers located at both shoulders, upper arms, lower arms, hands, upper legs, lower legs, feet, head, sternum, and pelvis. Data is wirelessly transmitted to a computer through Wi-Fi (IEEE 802.15.4). The accompanying software (Xsens MVN Studio version 4.2.1, Xsens Technologies B.V., Enschede, the Netherlands) allows to visualize and export full-body (consisting of 23 body segments) kinematics of the subject at the selected sampling rate of 240 Hz. The inertial sensors used in Xsens MVN provide about 1${}^{\circ }$ Root Mean Square Error (RMSE) in dynamic conditions and undisturbed magnetic field [6], whereas the joint angle accuracy of the system is in the range of 1 to 6${}^{\circ }$ [25]. It should be noted, that sensor orientation may differ from segment orientation due to soft-tissue artifacts, which was 3${}^{\circ }$ at largest for the knee joint angle [26].

A recording session starts with a simple calibration phase to estimate alignments between sensors and corresponding body segments. In this “N-pose” calibration, the subject is asked to stand upright, with arms next to body and palms facing the body, for a few seconds. For each tracking device, the system then fuses inertial and magnetic data to estimate sensor orientations which are further fed to another estimation engine that uses biomechanical constraints to estimate segment orientations and joint positions in a frame local to the laboratory (origin in the calibration position with x-axis aligned to magnetic North and z-axis upwards in the gravity direction) [1].

The proposed methods were validated using a reduced set of sensors, the orientations of considered individual sensors have been estimated using an Xsens tracking filter [27] and a calibration phase (N-pose) was performed as before to estimate corresponding segment orientations. Note that the use of a full-body motion capture system as an Xsens MVN allowed for the testing of different sensor configurations by just considering data from a subset of the 17 sensors.

### 2.4. Movement Database

All measured poses (about two million) define our movement database. Since movement data at 240 Hz resulted in almost indistinguishable adjacent poses, the movement database was down-sampled with a factor of 10, which resulted in approximately 200,000 poses. The size of the movement database is important as this impacts training (for ANN) and testing (for NNS) times of learning algorithms. In order to avoid dependencies from global orientations of the body in the environment, all segment orientations were expressed with respect to that of a reference body segment. The pelvis was chosen as the reference segment, because it typically experiences lower dynamics and is therefore measured with higher accuracy. Furthermore, in many activities (e.g., ADL) there is no evident coupling between movements of the upper and lower extremities [28]. By splitting full-body poses into two separate databases (containing upper- and lower-body segments, respectively), a larger number of full-body poses can be accounted for (even though never explicitly performed by any subject) by combining instances from the two databases.

### 2.5. Learning Process

**Figure 1:**
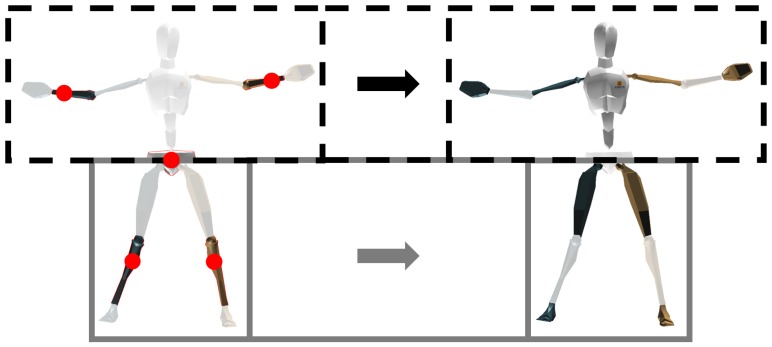
The left pose shows an example of input segment orientations (highlighted and sensor locations are marked with red dots) for both the upper- and lower-body estimations, whereas the right pose shows the corresponding output segment orientations (highlighted) for both the upper- and lower-body. Both estimations (displayed as black dotted and grey boxes) are combined to obtain the full-body pose.

The learning process (both for NNS and ANN) aims at estimating a full-body pose starting from the five known individual segment orientations. As described in Section 2.4, the unknown upper- and lower-body segment orientations are independently estimated starting from known segment orientations of upper- and lower-body, respectively. The full-body pose is derived by simply combining upper- and lower-body estimates. Many different sensor configurations (varying number and placement of sensors) could be considered. For the sake of clarity, in Figure 1 we provide an intuitive visualization of the process in the case of five sensors being used on the pelvis, lower arms, and lower legs, respectively. In this case, pelvis and lower legs measured orientations are used to estimate the rest of the lower-body segments, whereas pelvis and lower arms ones are used to estimate the rest of the upper-body segments. 

The focus of this work is to investigate the performance of the two learning paradigms (lazy and eager) at estimating full-body poses from low dimensional orientation features. Therefore, we chose to evaluate the performance of both ANN and NNS in a "snapshot" manner, where poses are estimated independently of each other, i.e., no temporal model that accounts for correlation between consecutive poses has been applied. Additionally, output of either algorithm was not explicitly corrected for poses implausible from a biomechanical viewpoint (e.g., knee flexion could result in angles larger than 180${}^{\circ }$).

The implementation of ANN was designed using the neural network toolbox of MATLAB R2016a (Mathworks, Inc., Natick, MA, USA). Function fitting networks (fitnet) were chosen for their regression capabilities of estimating non-linear relations between inputs and outputs, as the mapping of a few segment orientations to a full-body pose is assumed to be non-linear. Weight and bias values of the ANNs were updated using the scaled conjugate gradient backpropagation (trainscg). The networks were trained for a maximum of 1000 epochs and the training was finished if the gradient stopped decreasing for 6 epochs (i.e., a local minimum of the regression problem was found). The inputs to the neural network were orientations (expressed as quaternions) of the measured body segments, and the outputs were chosen to be the orientations of the remaining body segments. Each input/output neuron processed a single element of a quaternion, where the norm of the output was not explicitly enforced to be one. The neural network was expected to learn this from the training dataset, however, the output was normalized to one, such that this was ensured.

The NNS implementation was similar to that of Giuberti [24]. The in- and outputs were identical to that of the ANN implementation. The distance $\left(d_{n}\right)$ between measured orientation feature vectors and orientation feature vectors in the training dataset was computed using the mean quaternion shortest angle [29]:

(1) $$d_{n}=\frac{\sum_{s=1}^{S}2\cdot \arccos\left({\left[{\left(q_{s}\right)}^{-1}⊗q_{s}^{n}\right]}_{{}_{{}_{1}}}\right)}{S}$$

where $q_{s}$ is the quaternion describing the orientation of the input body segment *s* (with a total of *S* input body segments), *n* is the index in the training dataset (with a total of *N* poses), ⊗ denotes the quaternion multiplication, ${\left(■\right)}^{-1}$ is the quaternion inverse function and ${\left[■\right]}_{1}$ extracts the first component of the vector. The computed distances $\left(d_{n}\right)$ are used to compute a weighted pose, according to the following equation:

(2) $$\tilde{p}=\sum_{l=1}^{k}\left(w_{l}\cdot p_{l}\right)$$

where $p_{l}$ are poses in the training database, that are used to compute a weighted average full body pose ($\tilde{p}$) based on the *k* (neighbors) closest poses, where weights $\left(w_{n}\right)$ are defined as:

(3) $$w_{n}=\frac{max\left(d\right)-d_{n}}{\sum_{n=1}^{N}\left(max\left(d\right)-d_{n}\right)}$$

where d indicates a vector containing all calculated distances ($d_{n}$). The output orientations of pose $\tilde{p}$ were normalized to obtain proper unit quaternions, similar to the ANN implementation.

To avoid biased results, performance of both algorithms was tested independently on data from each subject (i.e., data from the same subject were never simultaneously appearing in training and testing). In particular, data from one subject was, in turn, used for testing, whereas data from the remaining five subjects was used for training. Furthermore, an n-fold cross-validation [30] was performed to determine optimal algorithm parameters. Similarly to before, randomly splitting data into training and validation datasets might result in data from the same subject occurring in both datasets, thus introducing bias. Therefore, we chose n=5 to allow us to split the training dataset subject-wise. Note that, since one subject is used, in turn, for testing, data from five subjects is left for training and cross-validation. The network configuration (for ANN) and the number of neighbors *k* (for NNS) were optimized in the cross-validation. For the sake of conciseness, the results of these cross-validations are presented in Table 2.

### 2.6. Performance Evaluation

Analyzing errors in the estimation of human motion is difficult, one has to deal with what people perceive as posed errors [31]. In similar works, either the Euclidean joint distance [15,24] or the joint angle errors [13,20] have been selected as error metrics. Furthermore, different applications may stress more errors on different parts of the body. A biomechanical application could focus more on specific joint angles, while a virtual reality application might be more interested in end-effector positions. For the sake of generality, both error metrics are reported and investigated in this work. In particular, joint angles are calculated as the relative orientation (to the proximal segment) of adjacent segments. The joint angle error (in three rotational directions) is then computed as the absolute difference between the measured and estimated joint angles. Joint positions are calculated using forward kinematics on segment lengths and orientations starting from the pelvis [32]. The joint position error is then computed as the Euclidean distance between the measured and estimated joint positions.

## 3. Results and Discussion

In this section, we evaluate the impact of sensor placement (Section 3.1) and different activities (Section 3.2) on the performance of NNS and ANN. Furthermore, generalization of such performance over different subjects (Section 3.3) and the impact of sensor noise (Section 3.4) are also investigated.

**Figure 2:**
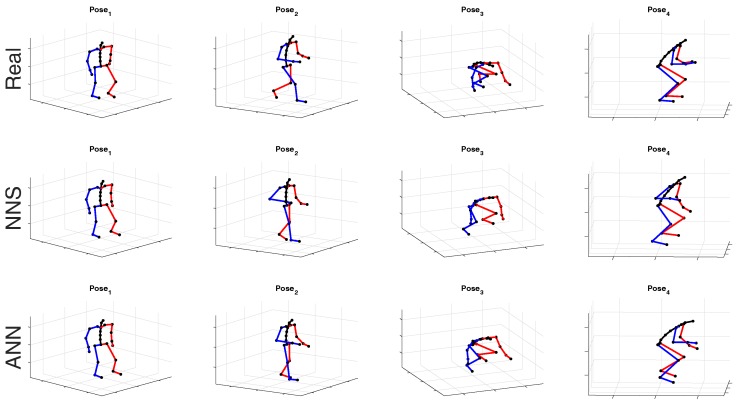
Measured and estimated poses of one subject are shown for different activities. Body segments on the left-side of the subject are colored red, while the right-side is colored blue. Joints are marked by black dots. The top row shows the measured poses of the testing dataset, the middle row shows poses estimated by NNS, and the bottom row shows those poses estimated by ANN (configuration D). Pose${}_{1}$ shows a pose in mid swing, pose${}_{2}$ is directly after toe off while carrying two cups, in pose${}_{3}$, a tray of cups is picked up and the subject is squatting in pose${}_{4}$.

Accompanying videos can provide the reader with a clear intuition of algorithms performance. Nonetheless, for lack of space, in Figure 2a representative selection of measured and estimated body poses for a few different activities (namely, walk, squatting, and kneeling) is shown.

### 3.1. Sensor Configuration Comparison

**Figure 3:**
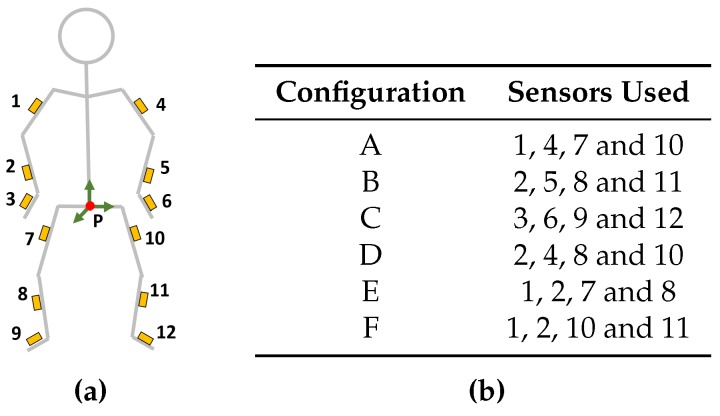
The different sensor configurations that have been investigated, each configuration is denoted by a letter (A–F) and the sensors for each of these configurations are numbered (1–12). (**a**) Sensor locations; (**b**) Sensors in the different configurations. In all configurations, the pelvis (P) is used as a reference segment.

Optimal sensor placement (on the body) in a reduced sensor system could be influenced by the requirements of the application of interest; in this work, we define it as the one that leads to the smallest average joint position/angle error. To limit the number of options, we investigated six configurations (all of them composed of five sensors), summarized in Figure 3a,b. As mentioned in Section 2.4, the pelvis (shown as the red dot marked with P) is chosen as the reference segment for all configurations. Since, in our approach, upper- and lower-body pose estimation is separately performed, the chosen configurations have been defined so that the sensors are split uniformly between upper- and lower-body.

**Figure 4:**
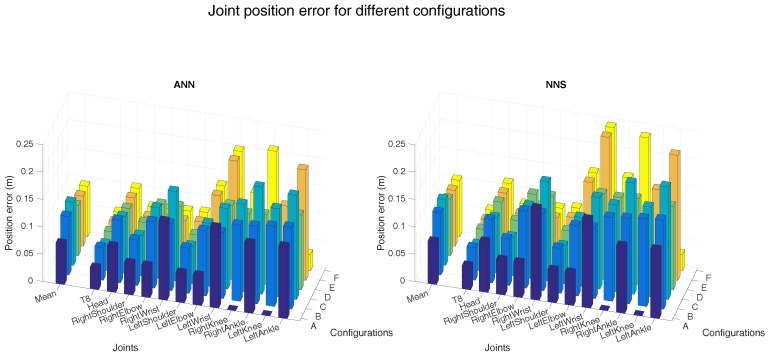
The left-side of both bar plots (ANN on the left and NNS on the right) shows the mean (over all six subjects) joint position error for the different configurations (as described in Figure 3). Individual mean (over all six subjects) joint position errors are shown on the right-side of both bar plots. A selection of joints is shown in both bar plots for readability. The different spine joints are not shown here because the joint position errors are comparable to the T8 joint. Additionally, the extra shoulder/foot joints are not presented because the magnitude of the error is similar to that of the shoulder and ankle joints shown.

**Figure 5:**
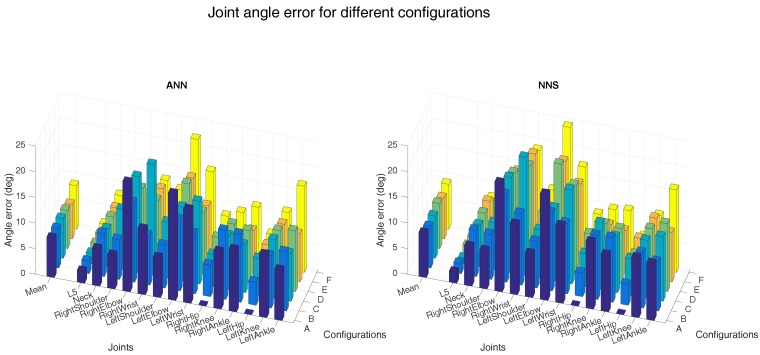
Mean (over all six subjects) joint angle errors for different configurations (as described in Figure 3) are shown on the left-side of both bar plots (ANN on the left and NNS on the right). Individual mean (over all six subjects) joint angle errors are shown on the right-side of both bar plots. A selection of joints is shown in both bar plots for readability. The different spine joints are not shown, because the joint angle errors are similar to the L5 joint. Similarly, extra shoulder/foot joints are not presented because the magnitude of the error is similar to that of the shoulder and ankle joints shown.

Figure 4 and Figure 5 show the joint position error and the joint angle error (averaged over all six subjects), respectively, for the different sensor placements. Performance of ANN and NNS are reported, by also detailing contribution of each segment/joint (groups of bars on the right-side of plots) to the mean error (single bars on the left-side of plots). In Figure 5, for the sake of conciseness, an average of joint angle errors, instead of errors in all three rotational directions (flexion/extension, abduction/adduction, and internal/external rotation), is shown. It can be observed that differences in performance between ANN and NNS are in the order of a few centimeters. The optimal sensor placement, according to the average joint position error, appears to be configuration A (namely, pelvis, upper legs, and upper arms) with 7 and 8 cm errors for ANN and NNS, respectively. These results are comparable to one of the reported situations in the work of Tautges et al. [15]. On the other hand, according to the average joint angle error, the optimal sensor placement is the one defined in configuration E (namely, an asymmetric configuration composed of pelvis, right upper and lower arm, right upper and lower leg) with errors equal to 7${}^{\circ }$ and 8${}^{\circ }$ for ANN and NNS, respectively.

Nonetheless, most of the considered configurations do not show major differences in average performance that would strongly motivate using one configuration over the remaining ones. Rather, it is important to highlight how errors on specific joints/segments are showing much larger variations. In that respect, it can be indeed noticed that average errors are mainly influenced by joint and segment errors which are particularly small due to the specific definition of the different configurations. For instance, configuration A shows shows hip angle errors of zero, because sensors are placed on adjacent body segments, consequently the knee joint position errors are zero.

In general, large individual joint position errors are shown at most distal joints (such as wrists and ankles) for all configurations, where errors could vary from 12 cm at the right ankle for configuration A (for NNS) to 24 cm at the left wrist for configurations E and F (for NNS). This is likely motivated by the fact that distal positions, since they are estimated using forward kinematics, tend to accumulate errors from segment to segment. On the other hand, joint angle errors show similar trends for different configurations. Even though these plots might better serve as tools for selecting optimal sensor placement in light of specific requirements of an application of interest, for the sake of a concise analysis, in the following we will investigate other aspects by choosing configuration D, which shows both mean joint angle and position errors close to the best found errors.

### 3.2. Activity Comparison

**Figure 6:**
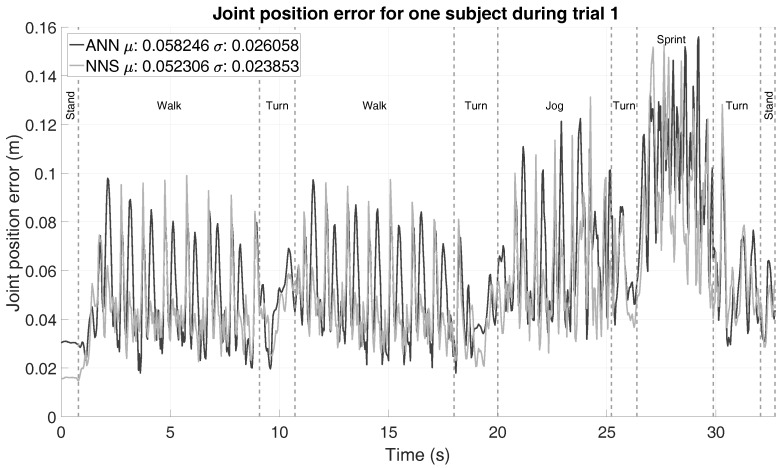
Average joint position error (using configuration D) of one subject during the first trial. Vertical dashed lines are shown to denote the different movements in that part of the trial.

So far, no focus has been put on investigating evolution of error over time. Therefore, an example of the joint position error progression over time (using configuration D) for trial 1 (as defined in Table 1) of one of the subjects is shown in Figure 6. Different events/activities in the trial are marked by vertical dashed lines and labeled accordingly. Beside observing the quick and frequent jumps in the joint position error, which is likely the result of the use of a snapshot approach (i.e., no connection between adjacent poses), it is quite evident that different activities show different (yet consistent for each activity) error trends. This emphasizes the importance of characterizing the impact of different activities on the algorithm’s performance.

By evaluating six different testing cases, as defined in Table 3, more insight into the performance of both ANN and NNS for different activities is provided. One trial for each activity was excluded from the training trials (of different subjects), such that capabilities of extrapolating movements could be analyzed.

**Figure 7:**
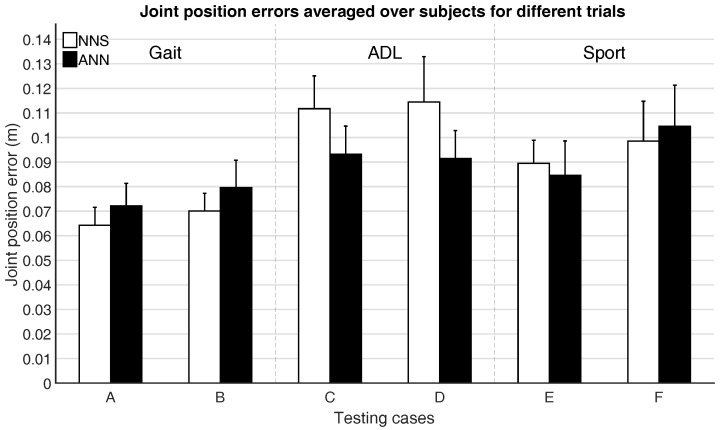
Mean (over all six subjects) and standard deviation (between the six subjects) of joint position errors for different testing cases, as described in Table 3.

**Figure 8:**
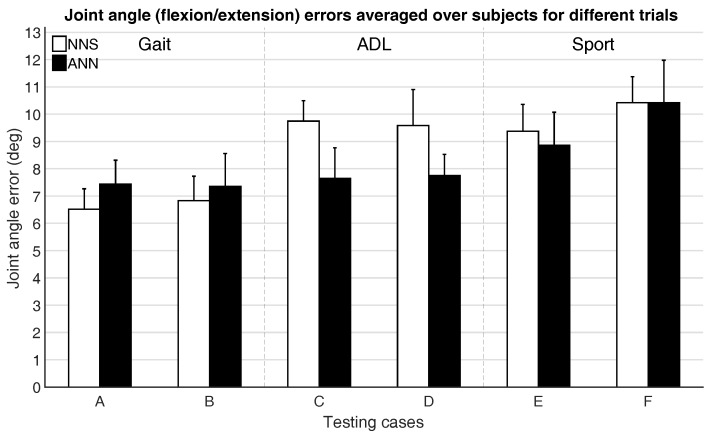
Mean (over all six subjects) and standard deviation (between the six subjects) of joint angle (flexion/extension) errors for different testing cases, as described in Table 3.

Figure 7 and Figure 8 show the mean joint position error and mean joint angle error (averaged over all subjects), respectively, for the different testing cases. Note that, in Figure 8, the average flexion/extension angle error is shown, as this is the rotational direction with the largest variability for most joints and often of most interest for biomechanical analysis.

Performance differences between activity classes in mean joint position and angle errors are a few centimeters and degrees. This might be part explained by the fact that for different activities similar poses can occur (e.g., standing, walking, etc.), thus favoring classes with the largest overlap in poses (such as gait, where walking poses are indeed likely also occurring in ADL and Sport).

Trials that were excluded from the training dataset (testing cases B, D, and F) are estimated with similar accuracy (differences are in the range of a few centimeters and degrees) as the included trials (testing cases A, C, and E), which might indicate that generalizing between different motions within activity classes is possible. In general, there are not clear evidences that would favor the choice of one learning approach over the other. A larger difference between ANN and NNS can be observed for ADL, compared to Gait and Sport activities. This difference, although in line with the empirical standard deviation range, might be explained with the capability of ANN of abstracting from the training database, while NNS depends on poses in the training database explicitly.

### 3.3. Generalizing Performance

**Figure 9:**
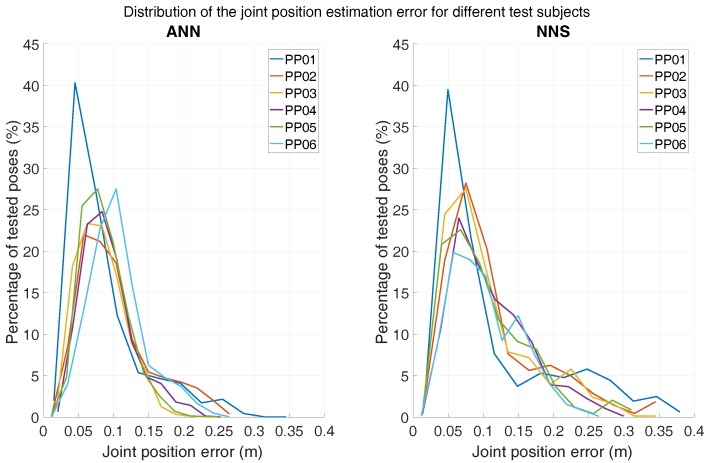
Distribution of joint position errors (with bins of 3 cm) for all scenario 1 activities for each subject. The left distribution shows ANN results, whereas NNS results are shown on the right.

Another aspect of interest is the difference in performance when testing over different subjects. In Figure 9, the distribution of the mean joint position error (over all trials) for each tested subject is shown, for both ANN and NNS. For ease of comparison, the distributions are overlapped and each tested subject is represented by a different color. The mode of the distributions for both ANN and NNS lies around 7 cm for all subjects and the whole distributions have similar shapes, indicating that the learning performances are quite generalizable over different subjects. Note that both ANN and NNS error distributions show long tails, which could be an indication of the (un)effectiveness of the algorithms at estimating rarely occurring poses in the movement database. Such error distribution profiles have also been reported by Tautges et al. [15].

### 3.4. Sensor Noise Analysis

Sensor orientation tracking from sensor fusion of IMU and magnetometers generally shows very small inclination errors and slightly larger heading errors, mostly due to the difficulty in determining magnetic North using magnetometers in the case of a magnetically disturbed environment. Although biomechanical body constraints can help in mitigating such errors if a full-body system is available, that may represent a harder challenge when using a reduced sensor set. During our data collection, we made sure that the environment was as clean as possible to guarantee the best quality in the collected data. Note that, this is even more important if such data are used as inputs for learning algorithms, as for our case. However, it is worth investigating the impact of noisy inputs on learning algorithms performance.

**Figure 10:**
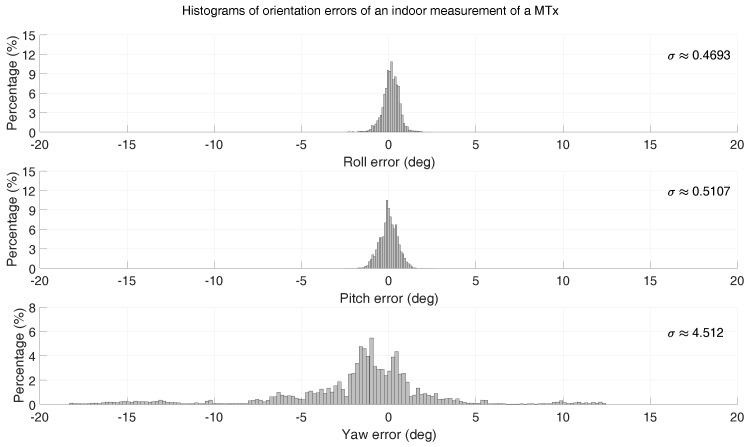
Orientational Root Mean Square Error (RMSE) errors of an Xsens motion tracker during a measurement of approximately 2 h.

To illustrate the scale of such orientation errors in a typical real-world scenario, a measurement of about 2 h was performed using an Xsens motion tracker identical to the ones used for the data collection in this work. This measurement was performed by a person carrying both sensors around in-/outside an office building (radiators, chairs, desks, and cars are examples of encountered sources of magnetic disturbances). A very accurate tactical grade IMU (0.75${}^{\circ }$/h gyroscope drift, 1 mg accelerometer resolution) was used as a reference to estimate the error of the sensor orientation measured by the Xsens motion tracker. Histograms of the RMSE error of roll, pitch, and yaw, respectively, are shown in Figure 10. As expected, it can be observed that the yaw (heading) error is much larger (σ≈4.51) than that of the inclination (roll/pitch) (σ≈0.47, σ≈0.51).

**Figure 11:**
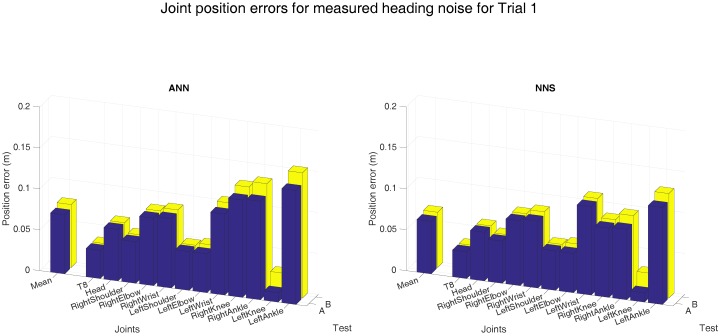
Mean (over all six subjects) joint position error obtained with ANN and NNS when the measured noise was applied to the heading orientation of the inputs. Here, A is the joint position errors obtained with the original input, whereas B is obtained with the measured heading errors applied to the original input.

Heading errors influence the measured orientation, however, its effects on the performance of learning algorithms remains unclear. Therefore, a white Euler rotation was applied to the measured sensor heading; the magnitude of this rotation was randomly drawn from the measured distribution (as shown in Figure 10). Note that, noise is overimposed only to testing data, since we do not want to corrupt the learned models. As our method uses a snapshot approach, a white signal is justified, since estimation errors are not a function of time. As expected, the joint position errors (averaged over all subjects) increase when noise is applied to the input (test B), as shown in Figure 11. Differences between tests A and B are in the range of 1 to 2 cm, whereas differences between both learning approaches are smaller than 1 cm.

#### Noise Sensitivity Analysis

**Figure 12:**
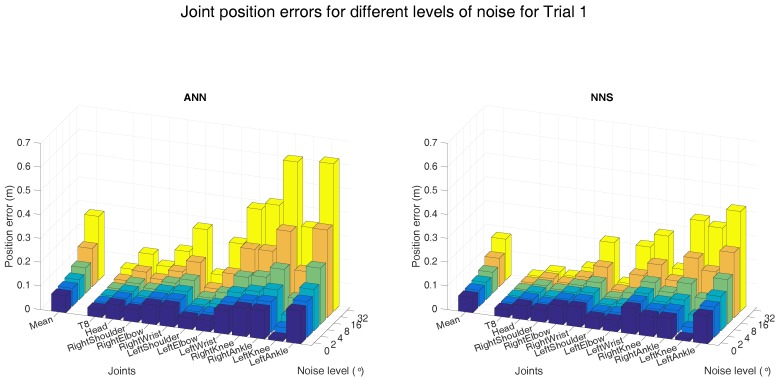
Mean (over all six subjects) joint position error obtained with ANN and NNS when a Gaussian white noise signal was applied to the heading orientation of the inputs. The magnitude of this rotation noise (standard deviation of the Gaussian white signal) is shown on the x-axis.

In order to further investigate robustness of both learning approaches, a sensitivity analysis was performed on the errors in the heading direction. To that end, we assumed that the measured heading error could be approximated by a Gaussian distribution. Simulated heading errors (with varying standard deviations, shown on the x-axis) were applied to the measured heading orientations, for which the resulting mean joint position errors are presented in Figure 12.

As expected, the mean joint position errors show an ascending trend for increasing levels of simulated noise. The 4${}^{\circ }$ simulated heading noise results are similar to those obtained with the actually measured heading errors, confirming the validity of the Gaussian assumption. At this noise level, the mean joint position error is 7 cm for NNS, while it is 8 cm for ANN. This difference is largely the result of the position error increase at the ankles, which is 17 cm for ANN, and 14 cm for NNS. This can be explained by the fact that ANN uses a trained model, which was not trained for data with noise and could therefore provide implausible poses as output. The estimated pose using NNS is more likely to be plausible, as it is a weighted average of poses in the training dataset. Therefore, NNS shows slightly smaller errors for increasing levels of noise on input orientations.

### 3.5. Computational Performance

In the introduction, (dis)advantages of the computational performance (training/testing time and storage size) of lazy and eager learning approaches were mentioned. To provide more insight into this performance, these values were calculated for a training database of 124,214 poses and a testing database of 32,060 poses, of which results are shown in Table 4. In this example, the configuration of the ANN was two hidden layers with 250 neurons in the first layer and 100 neurons in the second layer. NNS exploited 500 neighbors in this implementation.

As expected, a neural network estimates poses faster than NNS in the current implementation. The ANN results show potential for real-time estimation of full-body poses. An eager learning method, such as ANN, requires less storage space as the model is stored instead of the actual training data. As certain applications might require larger databases, the required storage might become an issue on a portable (embedded) system. Training time is less important if it is within reasonable boundaries, as training can be performed offline.

## 4. Conclusions

We have presented an in-depth performance analysis of ANN and NNS used for the estimation of full-body poses from orientations of a reduced set of IMUs (with magnetometers). The investigated approach showed a joint position error of approximately 8 cm and a joint angle error of approximately 7${}^{\circ }$. The obtained results did not show clear evidence of an algorithm outperforming the other (differences in joint position and angle errors were shown to be approximately 1 cm and 2${}^{\circ }$, respectively). Performance showed larger variations across different classes of activities, where smaller joint position/angle errors were obtained for gait, whereas ADL showed larger ones. Both algorithms have proved to be capable of generalizing over subjects. In a magnetic disturbed environment, NNS shows better performance (mean joint position error is 1 cm smaller) than ANN.

A choice for either algorithm would therefore depend on several factors, such as (but not limited to) computing power, real-time estimation, memory requirements, and/or magnetic disturbances. ANN is faster at run-time and requires less memory, but training times can be long and performance is poorer when magnetic interferences are corrupting the input signals. NNS is flexible (no model creation), with better performance in magnetic environments, but memory requirements can be large, as well as computation times (especially for large databases).

## 5. Future Work

Jittering between consecutive poses has been observed (see, for instance, Figure 6 in Section 3.2) in the estimated pose outputs. In the approaches discussed in this work, this is most likely the consequence of not considering past poses in the estimation of the current poses. Other works have used priors in a Bayesian approach to ensure smoothness in the pose estimations [13,15,16,20], which could be implemented in the current approach. An eager learning approach (such as a recurrent neural network) to predict a pose based on past poses could also be an interesting option. Additional information, such as biomechanical constraints, could be applied to improve pose estimation, as this would prevent implausible output poses, e.g., knee flexion angles cannot exceed 180${}^{\circ }$. Supplementary features, based for instance on sensor accelerations, might further improve current pose estimates.

Finally, specific target application requirements might help to focus the development of a reduced sensor motion capture system, because the resulting errors can be evaluated within its context. Possible applications could be in virtual reality, sports, and/or in health care. The current implementation outputs orientations/positions relative to the body. However, a specific application, such as virtual reality, might require global motion as an output. To that end, global position tracking could be implemented using the current sensors in combination with contact detection.

## Figures and Tables

**Table 1:** A description of trials in the experimental protocol (each trial was performed three times by all subjects). ADL = Activity of Daily Living, L = left and R = right.

	Trial	Short Description
Gait	1	Walk 10 m, walk 10 m, jog 10 m and sprint 10 m.
	2	Walk with a glass of water (dominant hand, non-dominant hand and in both hands)
	3	Walk 10 m, walk slowly 10 m, walk backwards 10 m, side-step six steps (L/R).
Sport	4	Two-legged jumps (4×), hops L/R (4×), run and jump L/R (2×), jump up (4×).
	5	Lunges L/R (4×), squats (4×), jumping jacks (4×).
	6	Sit-ups (5×) and side side-ups L/R (3×).
	7	Kick a ball against the wall L/R (3×).
	8	Throwing a ball against the wall L/R (3×).
	9	Crawling six steps.
ADL	10	Take a magazine, put it on the table, get seated, read a magazine, stand up and put it away.
	11	Take a tray with cups, walk with the tray, put it on the floor, stand up, pick it up.
	12	Take a glass, fill it with water and drink it in a chair.
	13	Put on a coat and take it off.
	14	Comb hair, scratch back, touch toes, rotate arms around shoulder back- and forward.
	15	Kneel down and tie shoelaces (L/R).
	16	Ascend and descend stairs.

**Table 2:** Optimization settings for different learning algorithms. Network configuration describes the number of neurons in the first and second hidden layer. *k* = the number of neighbors, NNS = Nearest Neighbor Search and ANN = Artificial Neural Network.

Learning Algorithm	Optimization Parameters	Value
NNS	*k*	500
ANN	Network configuration	[250 100]

**Table 3:** Description of the six testing cases, for the specified training database.

	Testing Case	Test Trial	Training Trials
Gait	A	1	1, 3, 4, 6, 7, 8, 9, 10, 12, 13, 14, 15 and 16
	B	2	
ADL	C	12	
	D	11	
Sport	E	4	
	F	5	

**Table 4:** Computation times (for a training database N=124,214) for both ANN and NNS using single-core computation on a Core i7 @ 2.5 GHz system with MATLAB R2016a. Training time is the total required time, run time is presented as an average per sample, and required storage is the total size (as MATLAB variables stored as double) of the trained neural network or the training database. * ANN was trained using parallel (4 cores) computation on the same pc, as this computation can be performed offline.

Learning Algorithm	Training Time (s)	Run Time (ms/Sample)	Required Storage (MB)
ANN	525.9 *	8.3	2.1
NNS	0	67.8	87.2

## Supplementary Materials

The Supplementary Materials are available online at http://www.mdpi.com/1424-8220/16/12/2138/s1.

## Abbreviations

The following abbreviations are used in this manuscript:

ADL	Activity of Daily Living
ANN	Artificial Neural Network
IMU	Inertial Measurement Unit
NNS	Nearest Neighbor Search
RMSE	Root Mean Square Error
